# Colonization of a hand washing sink in a veterinary hospital by an *Enterobacter hormaechei* strain carrying multiple resistances to high importance antimicrobials

**DOI:** 10.1186/s13756-020-00828-0

**Published:** 2020-10-21

**Authors:** Kanishka Kamathewatta, Rhys Bushell, Fannana Rafa, Glenn Browning, Helen Billman-Jacobe, Marc Marenda

**Affiliations:** 1 Department of Veterinary Biosciences, Faculty of Veterinary and Agricultural Sciences, The University of Melbourne, Werribee, VIC 3030 Australia; 2 Asia-Pacific Centre for Animal Health, Department of Veterinary Biosciences, Faculty of Veterinary and Agricultural Sciences, The University of Melbourne, Parkville, VIC 3052 Australia

**Keywords:** Veterinary hospital, ICU, Antimicrobial resistance, IncH12 plasmid, *Enterobacter hormaechei*

## Abstract

**Background:**

Hospital intensive care units (ICUs) are known reservoirs of multidrug resistant nosocomial bacteria. Targeted environmental monitoring of these organisms in health care facilities can strengthen infection control procedures. A routine surveillance of extended spectrum beta-lactamase (ESBL) producers in a large Australian veterinary teaching hospital detected the opportunistic pathogen *Enterobacter hormaechei* in a hand washing sink of the ICU. The organism persisted for several weeks, despite two disinfection attempts. Four isolates were characterized in this study.

**Methods:**

Brilliance-ESBL selective plates were inoculated from environmental swabs collected throughout the hospital. Presumptive identification was done by conventional biochemistry. Genomes of multidrug resistant *Enterobacter* were entirely sequenced with Illumina and Nanopore platforms. Phylogenetic markers, mobile genetic elements and antimicrobial resistance genes were identified in silico. Antibiograms of isolates and transconjugants were established with Sensititre microdilution plates.

**Results:**

The isolates possessed a chromosomal Tn7-associated silver/copper resistance locus and a large IncH12 conjugative plasmid encoding resistance against tellurium, arsenic, mercury and nine classes of antimicrobials. Clusters of antimicrobial resistance genes were associated with class 1 integrons and IS26, IS903 and ISCR transposable elements. The *bla*SHV-12, *qnrB*2 and *mcr*-9.1 genes, respectively conferring resistance to cephalosporins, quinolones and colistin, were present in a locus flanked by two IS903 copies. ESBL production and enrofloxacin resistance were confirmed phenotypically. The isolates appeared susceptible to colistin, possibly reflecting the inducible nature of *mcr*-9.1.

**Conclusions:**

The persistence of this strain in the veterinary hospital represented a risk of further accumulation and dissemination of antimicrobial resistance, prompting a thorough disinfection of the ICU. The organism was not recovered from subsequent environmental swabs, and nosocomial *Enterobacter* infections were not observed in the hospital during that period. This study shows that targeted routine environmental surveillance programs to track organisms with major resistance phenotypes, coupled with disinfection procedures and follow-up microbiological cultures are useful to control these risks in sensitive areas of large veterinary hospitals.

## Background

Hospital acquired infections are a significant threat to human and animal health. Hospital environments are critical reservoirs for drug-resistant bacteria [1–3]. Investigations into nosocomial infections outbreaks caused by *Enterobacterales*, *Pseudomonas* and *Acinetobacter*, have revealed that contaminated hand washing sinks in intensive care units were an important source of these microorganisms [4–8]. Dissemination of bacteria from the hand washing sinks is droplet-mediated [9]. Mobile genetic elements such as plasmids, integrons, and transposons play a key role in maintaining and propagating antibiotic resistance genes (ARGs). Common plasmid sequences have been detected in different species of carbapenemase-producing bacteria that colonised both the patients and the plumbing of an intensive care unit (ICU) in a human hospital [10]. Good biosecurity practices and routine, targeted environmental surveillance are two important tools to prevent outbreaks of nosocomial infections caused by multidrug resistant opportunistic or obligate pathogens in hospital premises. The genomic analysis of the bacteria isolated through these surveillance programs provides useful information on the origin and potential spread of antibiotic resistance genes. This knowledge can be used to improve infection control procedures. The aim of this study was to exploit the findings of a surveillance program for multi-drug resistant organisms in the environment of a teaching veterinary hospital. The genus *Enterobacter* represents a group of phylogenetically diverse opportunistic pathogens, often harboring multiple drug resistance genes and are involved in hospital-acquired infections [11]. Here, we report the repeated isolation of an extended spectrum beta lactamase (ESBL) producing, multidrug resistant *Enterobacter* sp. from the hand washing sink of a large veterinary hospital ICU, and its genotypic and phenotypic characterization. Antimicrobial resistance genes, integrons and transposons were identified in the isolates and their potential for mobility and horizontal transfer was investigated through comparative sequence analysis and conjugation experiments. A successful decontamination protocol was implemented in the hospital to eliminate the organism from the sink in response to these findings.

## Methods

### Bacterial isolates

Swabs from ICU sink and drain were submitted to the clinical microbiology laboratory of the Melbourne Veterinary School U-Vet hospital in Werribee, Victoria, Australia, as part of the routine environmental surveillance program. The swabs were placed in 100 ml of buffered peptone water (BPW) and incubated at 37 ºC for 24 h. ESBL screening plates (Oxoid) were inoculated with one loop of broth culture and incubated at 37 ºC for 24 h. Presumptively ESBL positive green or blue colonies were sub-cultured onto sheep blood agar and MacConkey agar (MicroMedia, Australia) plates, which were incubated at 37 ºC for 24 h. Phenotypic identifications were performed based on colony morphology, Gram staining characteristics, oxidase test and biochemical properties using the API rapidID 32E test (bioMerieux, Marcy-l'Étoile, France) and the Entero-Pluri test (Liofilchem) kits. ESBL production was confirmed with double disk diffusion synergy assays using cefotaxime, ceftazidime and amoxicillin-clavulanate [12]. Antimicrobial susceptibility testing was performed with the Calibrated Dichotomous Susceptibility method [13] and the broth microdilution method using Sensititre plates COMPGN1F and GNX2F (Thermo-Fischer) on a Aris2X machine according to the manufacturer’s instructions.

### DNA extraction

Single colonies from pure overnight cultures on sheep blood agar were inoculated into 10 ml of tryptic soy broth (TSB), which was incubated at 37 °C overnight. Cells from 1 ml of each TSB cultures were collected by centrifugation at 15,000×*g* for 2 min and genomic DNA was extracted using the Wizard Genomic DNA Purification Kit (Promega) according to the manufacturer’s protocol for Gram negative bacteria. The DNA concentration was measured using a Quantus fluorometer (Promega) and the quality was determined by microspectrophotometry (NanoDrop ND-1000, NanoDrop Technologies). The DNA extracts were cleaned using SPRI beads (AMPureX, Beckman Coulter).

### Nanopore sequencing

The sequencing libraries were prepared according to the 1D native barcoding genomic DNA sequencing protocol with EXP-NBD103 and SQK-LSK108 kits (Oxford Nanopore Technologies, Oxford, UK). At least 1 µg of DNA was processed by treatment with the Formalin-Fixed Paraffin-Embedded (FFPE) enzyme mix (New England Bio Labs, Ipswich, USA) and then end-repaired. Barcode-adaptor ligation was performed after dA-tailing. The sequencing was performed out in a MinION device with flow cell version FLO-MIN107 (Oxford Nanopore Technologies). The raw reads were basecalled into fastq files with Albacore version 2.2.7 (Oxford Nanopore Technologies). De-multiplexing and adaptor trimming was performed using Porechop version 0.2.3 (https://github.com/rrwick/Porechop), before filtering out 20% of the reads with the lowest quality, using the program Filtlong version 0.2.0 (https://github.com/rrwick/Filtlong).

### Illumina sequencing

Illumina sequencing was performed at the Australian Genome Research Facility (AGRF, Melbourne, Victoria, Australia) using the Illumina HiSeq2500 platform, generating 125 bp long paired-end reads. The sequencing adaptors were removed and the reads with a Phred quality score of < 20 were filtered out using Trim Galore version 0.4.4 [14].

### Genome assembly and analysis

Hybrid (short Illumina and long Nanopore reads) or long read-only de novo genomic assemblies were performed using Unicycler version 0.4.7 [15]. The identity of the genomes and their sequence type was determined using mlst (https://github.com/tseemann/mlst) within the PubMLST database [16]. The genomes and plasmids resulting from hybrid assemblies were annotated using the program Prokka version 1.14 [17] and the RAST annotation server [18]. Sequence visualization and plotting were performed with the Artemis program suite [19]. The annotations were manually curated using BLASTP to search the non-redundant protein database (NCBI). The antibiotic resistance genes were identified by searching the Prokka predicted open reading frames (ORFs) against the CARD protein database [20] with BLASTP. Transposons and integrons were predicted using ISfinder [21] and Integron Finder [22], respectively. The program IslandViewer [23] was used to visualise genomic islands. The origins of transfer regions on plasmids were identified using oriTfinder [24]. Multilocus Sequence Type (MLST) and Ribosomal Multilocus Sequence Typing (rMLST) analysis were performed on the pubMLST server [25, 26]. The program ABRicate [27] version 0.9.8 was used to detect antimicrobial genes with the databases ncbi and card, and incompatibility groups with the database plasmidfinder.

### Comparative sequence analysis

Full genome and plasmid alignments were performed using Mauve aligner version 2.4.0 [28]. Detailed single nucleotide polymorphism (SNP) and gap analysis of genome and plasmid alignments were performed using Geneious version 11.1.2. The plasmid sequences were searched against the NCBI nucleotide database and the PLSDB plasmid database [29] using BLASTN. Comparative plasmid visualizations were performed using the genoPlotR [30] package in R version 3.4.0. and the CGView Comparison Tool [31]. Phylogenetic trees were built with MegaX software [32] from concatenated multiple alignments of housekeeping gene sequences produced with the program Muscle [33].

### Mating

Broth mating experiments were performed as described before [34, 35]. Briefly, the recipient *E. coli* DH5ɑ and the donor CM18-216 were inoculated into 1 mL of LB and grown respectively at 37 °C (recipient) and either 27 °C or 37 °C (donor) for 18–20 h without shaking. One volume of donor cultivated at either 27 °C or 37 °C was mixed with four volumes of recipient and incubated at the same temperature for 2 h. Subsequently, 100 μl of the conjugation mixtures were plated onto LB agar plates containing 16 μg/ml tetracycline and 16 μg/ml nalidixic acid, and incubated at 27 °C and 37 °C for 48 h and 24 h, respectively. Colonies were randomly picked and sub-cultured on Sheep Blood Agar and MacConkey Agar plates for phenotypic testing.

## Results

### Persistence of a multidrug resistant strain of *Enterobacter hormaechei* in a sink

Four ESBL producing *Enterobacter* sp. were isolated on selective media from environmental swabs collected in a veterinary teaching hospital ICU over a period of approximately one month. The first isolate, CM18-216, was obtained from a hand-washing sink as part of the hospital routine surveillance program. A second isolate, CM18-242-2, was obtained from a follow-up assessment of the tap handles and sink edges after a first disinfection attempt. Two more isolates, namely CM18-269-1 and CM18-269-2, were later recovered from the drain and the edge of the same sink, after a second disinfection attempt. All isolates had identical antimicrobial resistance profiles.

The biochemical characterisation of isolates CM18-216 and CM18-242-2 established with the rapid ID 32E strip (Bio-Merieux) resulted in the profile 46772514741, which the ApiWeb database reports as an excellent identification of an *Enterobacter cloacae* (%ID 99.9, T 0.97). This identification was confirmed by an Entero-pluri test, giving the biocode 32261. However, an atypical result, Lactose negative, was indicated by the test. Moreover, the sink isolates did not ferment lactose on MacConkey plates.

The genomes of isolates CM18-216 and CM18-242-2 were completely sequenced, using Illumina and Oxford Nanopore platforms. A summary of sequence read statistics for both methods is provided in Additional file 1: Table S1. Isolate CM18-216 contained a 4,689,992 bp chromosome and a 288,096 bp plasmid; isolate CM18-242-2 contained a 4,689,986 bp chromosome and a 288,061 bp plasmid. The two isolates had nearly identical chromosome sequences, with only 44 single nucleotide differences and 6 small insertion/deletions which accounted for the 6 bp length difference (Table 1). These nucleotide differences were all in hypothetical proteins or in non-coding regions, except for one position within a 16S rRNA region. Several prophages and genomic islands were also identified on the chromosome (Additional file 2: Fig. S1). The sequence alignments of the large plasmids, hereafter named pCM18-216 and pCM18-242-2, revealed 16 nucleotide differences between the two sequences, as well as 6 gaps in pCM18-216 and 41 gaps in pCM18-242-2 (Table 2). These nucleotide differences were all clustered in a region of approximately 380 bp encoding an IS5 family transposase. The plasmids belonged to the incompatibility group IncHI2 and contained all the genes required for transfer and an oriT region, indicating that it was capable of conjugation.

**Table 1:** Nucleotide differences identified between the chromosomes of isolates CM18-216 and CM18-242-2

Position		Nucleotide difference	Protein/region
CM18-216	CM18-242-2		
308741	308741	G/A	Hypothetical protein
308748	308747	C/T	Hypothetical protein
309078	309077	G/A	Non-coding region
309785	309784	T/A	Non-coding region
309787	309786	C/T	Non-coding region
424216	424215	T/A	Hypothetical protein
425369	425368	G/A	Non-coding region
426076	426075	T/A	Non-coding region
426078	426077	C/T	Non-coding region
426577	426576	C/T	Non-coding region
1201725	1201724	C/T	16S rRNA
1203869	1203868	T/A	Hypothetical protein
1204685	1204684	G/A	Hypothetical protein
1204692	1204690	C/T	Hypothetical protein
1205022	1205020	G/A	Non-coding region
1205729	1205727	T/A	Non-coding region
1205731	1205729	C/T	Non-coding region
3657012	3657010	G/A	Non-coding region
3657014	3657012	A/T	Non-coding region
3657721	3657719	C/T	Non-coding region
3658051	3658049	G/A	Hypothetical protein
3658058	3658055	C/T	Hypothetical protein
3658874	3658871	A/T	Hypothetical protein
4172066	4172063	G/A	Non-coding region
4172068	4172065	A/T	Non-coding region
4172775	4172772	C/T	Non-coding region
4173928	4173925	A/T	Hypothetical protein
4437299	4437296	G/A	Non-coding region
4437301	4437298	A/T	Non-coding region
4438008	4438005	C/T	Non-coding region
4438338	4438335	G/A	Hypothetical protein
4438345	4438341	C/T	Hypothetical protein
4439161	4439157	A/T	Hypothetical protein
4515406	4515402	G/A	Non-coding region
4515408	4515404	A/T	Non-coding region
4516445	4516441	G/A	Hypothetical protein
4516452	4516447	C/T	Hypothetical protein
4517268	4517263	A/T	Hypothetical protein
4640694	4640689	G/A	Non-coding region
4640696	4640691	A/T	Non-coding region
4641403	4641398	C/T	Non-coding region
4641733	4641728	G/A	Hypothetical protein
4641740	4641734	C/T	Hypothetical protein
4642556	4642550	A/T	Hypothetical protein
308742	308742	T/-	Hypothetical protein
1204686	1204685	T/-	Hypothetical protein
3658054	3658052	A/-	Hypothetical protein
4438341	4438338	A/-	Hypothetical protein
4516448	4516444	A/-	Hypothetical protein
4641736	4641731	A/-	Hypothetical protein

**Table 2:** Nucleotide differences identified between the plasmids pCM18-216 and pCM18-242-2

Position		Nucleotide difference	Protein/region
pCM18-216	pCM18-242-2		
22872	22870	T/G	IS5 family transposase
22873	22871	T/C	IS5 family transposase
23000	22975	A/G	IS5 family transposase
23029	23004	A/G	IS5 family transposase
23080	23047	A/T	IS5 family transposase
23081	23048	C/G	IS5 family transposase
23111	23076	A/G	IS5 family transposase
23117	23082	A/C	IS5 family transposase
23166	23129	G/A	IS5 family transposase
23181	23144	G/A	IS5 family transposase
23183	23146	G/T	IS5 family transposase
23184	23150	C/A	IS5 family transposase
23185	23151	A/G	IS5 family transposase
23254	23219	T/C	IS5 family transposase
23255	23220	G/A	IS5 family transposase
218820	218785	G/A	IS5 family transposase
22869	22869	CT/–	IS5 family transposase
22889	22887	TTCCGA/------	IS5 family transposase
22924	22916	-/A	IS5 family transposase
22950	22943	T/-	IS5 family transposase
22968	22960	CGGATTAACCCGTTCCT/-	IS5 family transposase
23054	23029	TG/--	IS5 family transposase
23066	23039	GCGCTT/------	IS5 family transposase
23085	23052	A/-	IS5 family transposase
23093	23059	T/-	IS5 family transposase
23118	23083	-/T	IS5 family transposase
23157	23123	CAG/---	IS5 family transposase
23183	23146	---/ACC	IS5 family transposase
23242	23208	CC/--	IS5 family transposase
23253	23217	-/A	IS5 family transposase

The genomes of the two other isolates subsequently recovered from the ICU sink, CM18-269-1 and CM18-269-2, were sequenced with Nanopore reads only, and were assembled into 2 circular contigs corresponding to a chromosome and a plasmid. Multiple sequence alignments showed high levels of colinearity between all chromosomal contigs, suggesting that all four *Enterobacter* isolates recovered from the ICU sink over one month were related. However, while all four genomes carried a complete prophage of approximately 32 kb located 1160 kb from the chromosomal origin, the isolate CM18-269-2 possessed a second prophage, which was absent from the 3 other genomes, 1800 kb apart (Additional file 3: Fig. S2).

**Fig. 1:**
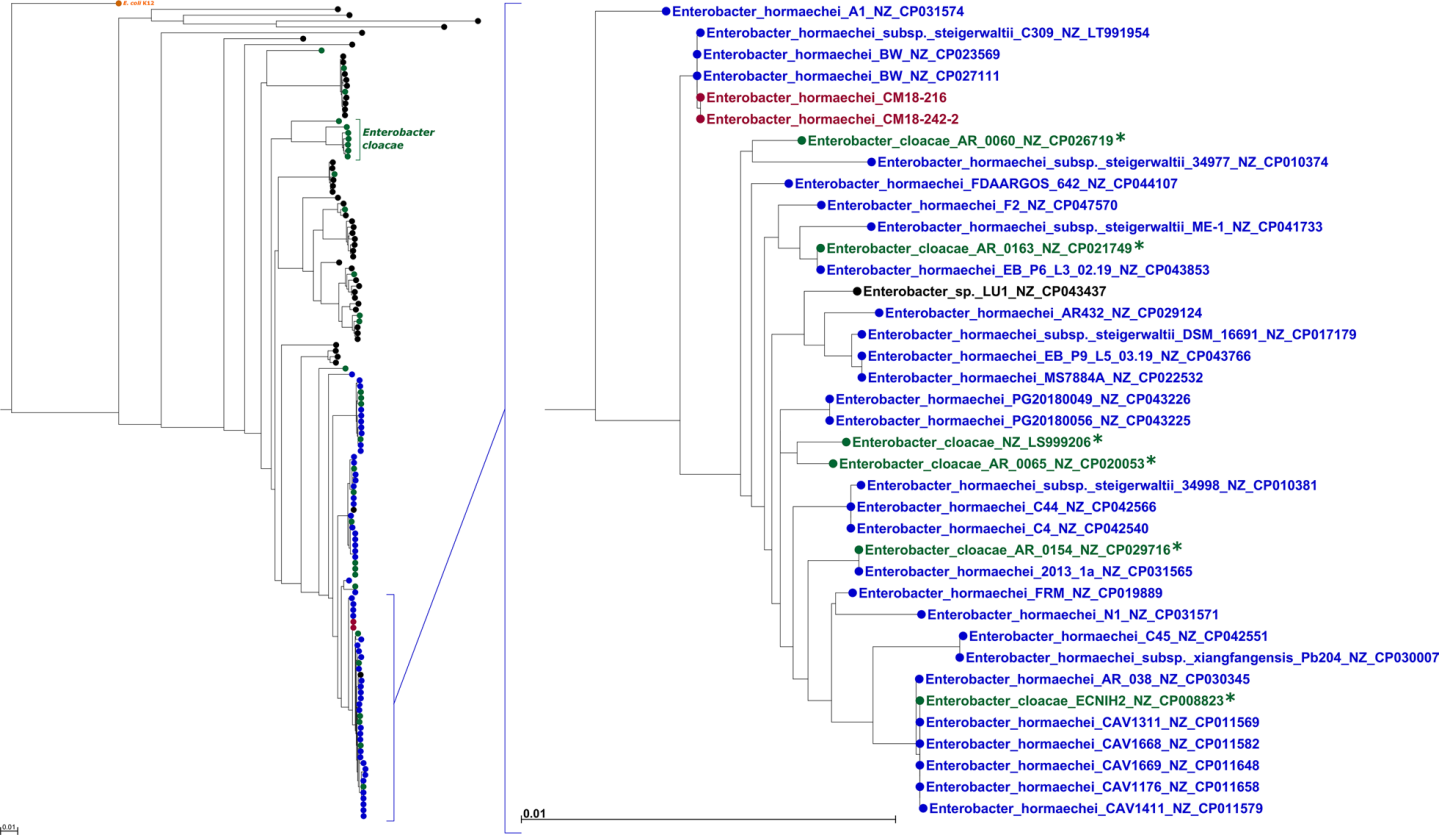
Phylogenetic analysis of 136 *Enterobacter* sp. genomes from RefSeq and the two sink isolates CM18-216 and CM18-242-2. A maximum likelihood tree was built from concatenated alignments of *groL, gyrA*, *gyrB, rpoB* and *dnaA* genes, representing 12174 positions, using the General Time Reversible model with discrete gamma distribution G (5 categories, 0.5920) and invariable sites I (58.19%). The *E. coli* K12 MG1655 strain is used as an out-group (orange). The *E. hormaechei*, *E. cloacae*, other *Enterobacter* sp., and sink isolates are respectively indicated in blue, green, black and red. The sub-tree containing the sing isolates is presented on the right. The asterisks indicate *E. cloacae* genomes re-classified as *E.* *hormaechei* by rMLST analysis. Scale bars represent the number of substitutions per site

The Sequence Type of CM18-216 was determined by the online pubMLST server as ST110, using the *Enterobacter cloacae* database. Ribosomal Multilocus Sequence Typing (rMLST) genome analysis identified CM18-216 and CM18-242-2 as *E. hormaechei*. Average Nucleotide Identity (ANI) analysis confirmed this result, indicating a higher proximity with *E. hormaechei* than with *E. cloacae* type strains (Table 3). Phylogenetic analysis of concatenated alignments of the house keeping genes *groL, gyrA*, *gyrB, rpoB* and *dnaA* from 142 *Enterobacter* sp. complete genomes from RefSeq (Additional file 1: Table S2) placed the two sink isolates on the same branch, amongst a cluster of *E. hormaechei* strains (Fig. 1). Of note, some entries identified as “*E. cloacae*” in the Ref_Seq database which were used to build the tree also fell into *E. hormaechei* clades. These genomes were individually analysed by rMLST, which re-classified them as *E. hormaechei*. The isolate CM18-216 was selected as representative of the *E. hormaechei* strain repeatedly found in the hospital ICU sink.

**Table 3:** ANI analysis of CM18-216, *E. cloacae* and *E. hormaechei* type strains

ANIm value (aligned percentage)	CM18-216	*Enterobacter cloacae* ATCC 13047 [T]	*Enterobacter hormaechei* ATCC 49162 [T]
CM18-216	*	87.97 (78.21)	95.30 (83.74)
*Enterobacter cloacae *ATCC 13047 [T]	87.97 (70.88)	*	87.89 (69.12)
*Enterobacter hormaechei* ATCC 49162 [T]	95.31 (85.51)	87.89 (77.99)	*

### Resistance genes to clinically important antimicrobials and to heavy metals are clustered on the large IncH12 plasmid

**Fig. 2:**
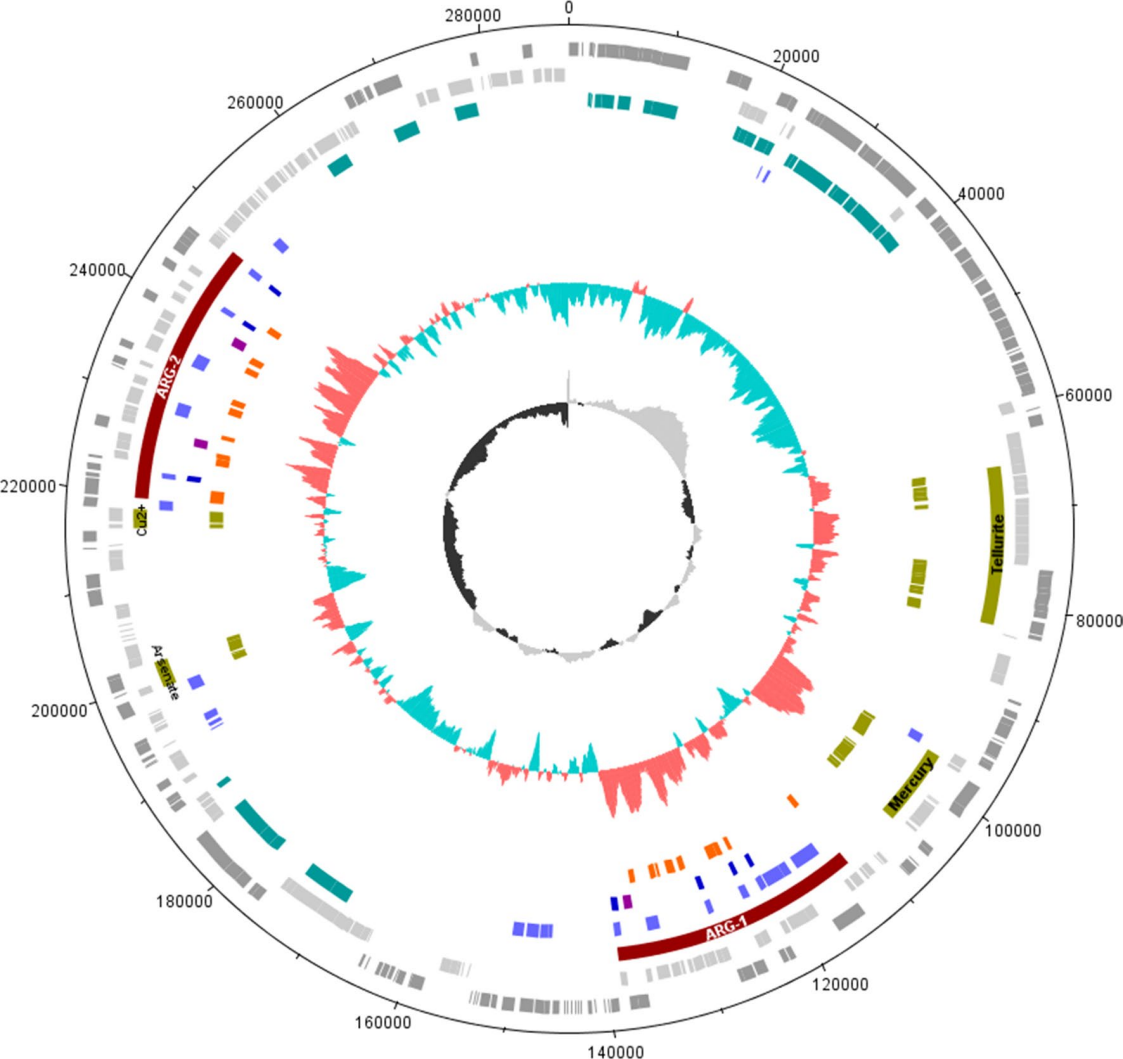
Genetic map of the plasmid pCM18-216. From outer to inner circles: 1-nucleotide positions; 2,3-grey bars: CDSs; 4-teal: conjugation, dark red: ARG loci, green–brown metal/other resistance loci; 5-light blue: transposases CDSs; 6-dark blue: IS26 elements, purple: integron recombinase CDSs; 7-orange: ARGs, green–brown: other resistance genes; 8-GC% plot; 9-GC skew plot

Thirty-five ARGs were detected on *E. hormaechei* CM18-216 genome by the program ABRicate, with 18 on the chromosome and 16 on the IncH12 plasmid (Table 4). The clinically important ARGs were clustered within two loci on the plasmid (Fig. 2). These ARGs were identified as *bla*SHV-12, *qnrB2*, *mcr*-9.1, *bla*-TEM, *cat*II, *tetD*, *sul*1, *dfrA*19, *ereA*, *arr*, *aac*(3)-II, *aac*(6′)-IIc, *aph*(6)-Id, *aph*(3″)-Ib and *ant*(3″)-Ia.

**Table 4:** Antimicrobial resistance genes in *Enterobacter* CM18-216

Gene	Start	End	Strand	% Cov	% Id	Resistance
Chromosome						
*bacA*	652522	653339	+	99.51	83.37	Peptide
*emrB*	1048287	1049816	−	99.42	84.25	Fluoroquinolone
*emrR*	1051153	1051683	−	100	84.18	Fluoroquinolone
*oqxA*	1315626	1316800	+	99.91	87.08	Phenicol Quinolone
*oqxB*	1316824	1319943	+	98.95	89.14	Phenicol Quinolone
*acrD*	1356278	1359376	−	99.52	81.41	Aminoglycoside
*yojI*	1537237	1538876	+	99.64	78.93	Peptide
*baeR*	1652616	1653326	−	98.34	82.98	Aminocoumarin Aminoglycoside
*mdtC*	1656139	1659216	−	99.94	82.44	Aminocoumarin
*mdtB*	1659217	1662339	−	99.9	80.23	Aminocoumarin
H-NS	2063229	2063642	−	100	85.51	Cephalosporin Fluoroquinolone Macrolide Penam Tetracycline
*marA*	2349683	2350058	+	97.92	84.84	Various
*msbA*	2956274	2958022	−	100	83.25	Nitroimidazole
*acrA*	3457822	3459015	+	100	87.94	Various
*acrB*	3459038	3462184	+	99.87	85.02	Various
*fosA*	3985418	3985843	−	100	96.01	Fosfomycin
CRP	4226794	4227426	+	100	87.99	Fluoroquinolone Macrolide Penam
*cpxA*	4567511	4568877	+	99.49	83.17	Aminocoumarin Aminoglycoside
Plasmid						
*bla*TEM-1	112126	112986	−	100.00	100.00	Beta-lactam
*catA*2	122343	122984	+	100.00	100.00	Chloramphenicol
*tet*(D)	124596	125780	+	100.00	99.92	Tetracycline
*sul*1	128882	129721	−	100.00	100.00	Sulfonamide
*ere*(A)	130245	131304	−	86.31	99.44	Macrolide
*arr*	132166	132579	−	100.00	100.00	Rifamycin
*aac*(3)-II	132707	133516	−	100.00	100.00	Gentamicin
*aac*(6′)-IIc	135601	136182	−	100.00	100.00	Gentamicin Kanamycin Tobramycin
*mcr*-9.1	219313	220932	+	100.00	100.00	Colistin
*aph*(6)-Id	224139	224975	−	100.00	100.00	Streptomycin
*aph*(3″)-Ib	224975	225777	−	99.88	100.00	Streptomycin
*dfrA*19	227552	228121	−	100.00	100.00	Trimethoprim
*sul*1	230833	231672	−	100.00	100.00	Sulfonamide
*qnrB*2	232163	232807	+	100.00	100.00	Quinolone
*sul*1	236554	237393	−	100.00	100.00	Sulfonamide
*aadA*2	237898	238689	−	100.00	100.00	Streptomycin
*bla*SHV-12	242434	243294	+	100.00	100.00	Cephalosporin

% Cov.: percentage of coverage; % Id.: percentage of identity

Phenotypic testing of the isolates broadly confirmed the resistance patterns predicted by genetic analysis. The isolates CM18-216 and CM18-242-2 possessed high Minimal Inhibitory Concentrations (MICs) values for penicillins, cephalosporins, monobactams, aminoglycosides, phenicols, trimethoprim-sulfonamides and tetracyclines (Table 5). Analysis of MICs for third generation cephalosporins and double-disk diffusion synergy assays confirmed the ESBL phenotype seen on selective plates during the primary isolation of the organism from environmental swabs. The MIC for enrofloxacin of both isolates was 1 ug/mL. The organism displayed a susceptible phenotype to colistin and polymyxin B in broth and agar diffusion tests.

**Table 5:** MIC of *Enterobacter* sink isolates, DH5 alpha transconjugant (TG) and parental recipient strain used in mating experiments

Antimicrobic	*E.* *hormachei* 18-216	*E. hormachei* 18-242-2	DH5 alpha_TG	DH5 alpha
Amikacin	≤ 4	≤ 4	≤ 4	≤ 4
Amoxicillin/Clavulanic Acid	> 8	> 8	= 8	= 4
Ampicillin	> 8	> 8	> 8	= 2
Aztreonam	> 16	> 16	> 16	n/d
Cefalexin	> 16	> 16	= 16	= 4
Cefazolin	> 32	> 32	= 32	= 2
Cefepime	≤ 2	≤ 2	≤ 2	n/d
Cefotaxime	= 8	= 8	= 2	n/d
Cefovecin	> 8	> 8	= 8	= 0.5
Cefpodoxime	> 8	> 8	= 8	≤ 1
Ceftazidime	> 16	> 16	= 16	≤ 4
Chloramphenicol	> 32	> 32	> 32	≤ 2
Ciprofloxacin	≤ 0.25	≤ 0.25	≤ 0.25	n/d
Colistin	≤ 0.25	≤ 0.25	≤ 0.25	n/d
Doripenem	≤ 0.12	≤ 0.12	≤ 0.12	n/d
Doxycycline	> 16	> 16	= 16	= 0.5
Enrofloxacin	= 1	= 1	≤ 0.12	≤ 0.12
Ertapenem	≤ 0.25	≤ 0.25	≤ 0.25	n/d
Gentamicin	> 8	> 8	> 8	≤ 0.25
Imipenem	≤ 1	≤ 1	≤ 1	≤ 1
Levofloxacin	≤ 1	≤ 1	≤ 1	n/d
Marbofloxacin	= 0.5	= 0.5	≤ 0.12	≤ 0.12
Meropenem	≤ 1	≤ 1	≤ 1	n/d
Minocycline	= 16	= 16	= 8	n/d
Orbifloxacin	= 4	= 4	≤ 1	≤ 1
Piperacillin/tazobactam constant 4	≤ 8	≤ 8	≤ 8	≤ 8
Polymixin	≤ 0.25	≤ 0.25	≤ 0.25	n/d
Pradofloxacin	= 0.5	= 0.5	≤ 0.25	≤ 0.25
Tetracycline	> 16	> 16	> 16	≤ 4
Ticarcillin/clavulanic acid constant 2	= 32	= 32	= 32	n/d
Tigecycline	= 0.5	= 0.5	= 0.5	n/d
Tobramycin	= 8	> 8	= 2	n/d
Trimethoprim/sulfamethoxazole	> 4	> 4	> 4	≤ 0.5

The values are compiled from Sensitre plates COMPGN1F and GNX2F. n/d: no data available for the organism

Gene operons or clusters for tellurium, mercury and arsenic metal resistance were also detected on the plasmid (Fig. 2). The tellurium resistance gene cluster was located between nucleotide positions 76066 and 82286, and consisted of *terZ, terA, terB, terC, terD, terE* and *terF*. The components of the mercury resistance operon, *merE, merD, merA, merC, merP, merT* and *merR*, were located between nucleotide positions 101821 and 105561. The arsenic resistance operon contained *arsH, arsR, arsB* and *arsC* and was located between nucleotide positions 199610 and 201790. The operon was co-located with an ISNCY family transposase, to the left of *arsH*. In addition to these plasmid operons, two complete copper and silver resistance loci, *pcoABCDRSE* and *silESRCFBAP* were present on the chromosome, next to Tn7-like transposases, in a predicted genomic island located between nucleotide positions 4356976 and 4393429 (Additional file 2: Fig. S1).

### The multidrug resistance plasmid pCM18-216 is conjugative

Mating between *E. hormaechei* CM18-216 and a laboratory strain of *E. coli* DH5ɑ (lactose negative, nalidixic acid resistant) in broth at 27 °C for 2 h resulted in the appearance of tetracycline-resistant transconjugants, which were confirmed as the *E. coli* recipient by conventional biochemistry. Mating performed at the higher temperature of 37 °C did not result in transconjugants.

The MICs of four randomly picked transconjugants were compared to the *E. hormaechei* and *E. coli* DH5ɑ parents (Table 5). All transconjugants had MICs identical to the donor and higher than the recipient for ampicillin, chloramphenicol, gentamicin, tetracycline, and trimethoprim-sulfamethoxazole. Moreover, the transconjugants had MICs higher than DH5ɑ, albeit slightly lower compared to the donor, for amoxicillin/clavulanic acid, first and third generation cephalosporins (cefalexin, cefazolin, cefovecin, cefpodoxime, ceftazidime), and doxycycline. However, the tranconjugants MICs for fluoroquinolones were similar to the unconjugated DH5ɑ recipient, and lower than the *E. hormaechei* donor.

### Antimicrobial resistance genes are associated with transposable elements

The two antibiotic resistance gene loci carried by plasmid pCM18-216 contained transposons and/or class 1 integrons putatively forming complex transposable elements.

**Fig. 3:**
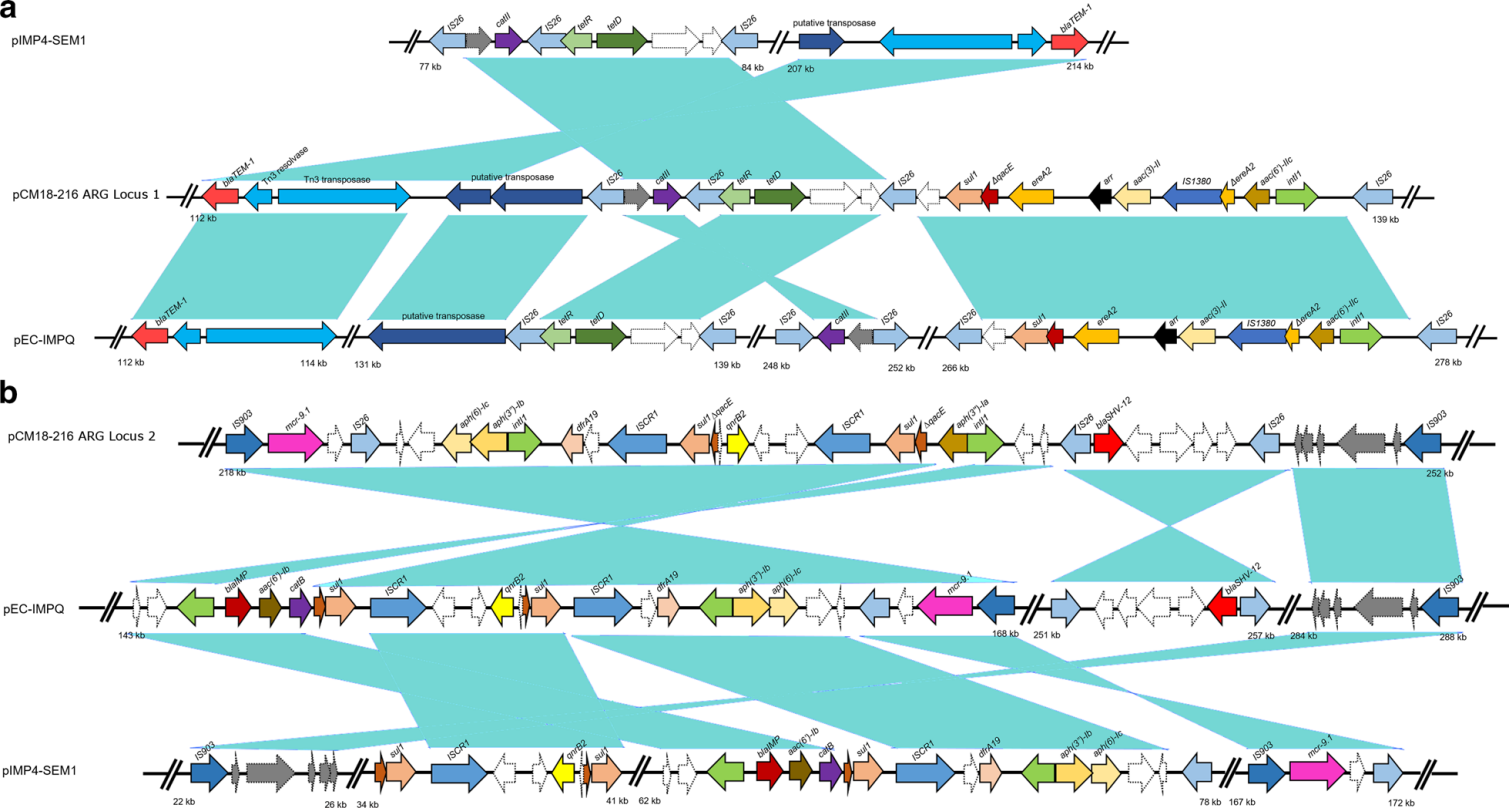
Schematic representation of the genetic context of the IS26 transposable elements in pCM18-216 and comparison with related plasmids pEC-IMPQ (NC_012556.1) and pIMP4-SEM1 (KX810825.1). Resistance genes and mobile elements are represented by color-coded arrows. **a** ARG locus 1; **b** ARG locus 2

Locus 1 was identified as an 18 kbp fragment consisting of two composite transposons and a class 1 integron fused together. The locus contained four IS26 copies, with the chloramphenicol resistance gene *cat*II between the first two, the tetracycline resistance gene *tet*D and its regulator *tet*R between the second and third, and a complete class 1 integron between third and fourth IS26 elements. The integron contained the aminoglycoside resistance gene *aac*(6′)-IIc upstream of the integrase gene, an IS1380 family transposase gene, and the aminoglycoside, rifampicin and erythromycin resistance genes *aac*(3)-II, *arr* and *ere*A, between the transposase and the 3′-CS of the integron. This structure appears to be the result of genetic re-arrangements involving IS26 family composite transposons conferring chloramphenicol and tetracycline resistance, together with a class 1 integron carrying the other resistance genes. This brought together 7 complete and 2 truncated ARGs that potentially could be mobilised in a single horizontal gene transfer event. Moreover, a beta-lactamase gene *bla*-TEM associated with a Tn3 transposon was located at the end of locus 1. These various components were also detected in plasmids with high levels of sequence similarity with pCM18-216, exemplified by pEC-IMPQ (NC_012556.1) carried by an *Enterobacter* isolated from a hospital environment in Taiwan, and pIMP4-SEM1 (KX810825.1) carried by a *Salmonella* isolated from a cat in Australia. However, the different genetic elements forming the pCM18-216 ARG locus 1 were located in separate regions in those replicons (Fig. 3a).

Locus 2 was a 26 kbp structure, also containing IS26 elements. The region is bordered by two IS903 copies and carries composite transposons and a complex class 1 integron containing two integrase genes, surrounding two “insertion sequence common region 1” elements (ISCR1, or IS91 family transposases). Eight ARGs were found in locus 2, including the ESBL *bla*SHV-12, fluoroquinolone resistance *qnrB*2 and colistin resistance *mcr*9.1, which were respectively associated with copies of IS26, ISCR1 and IS903. As for locus 1, these structures were also found in pEC-IMPQ and pIMP4-SEM1 but were organized differently and carried a slightly larger repertoire of ARGs (Fig. 3b).

### pCM18-216 shows similarities with a subset of large multidrug resistance plasmids from *Enterobacteriaceae*

**Fig. 4:**
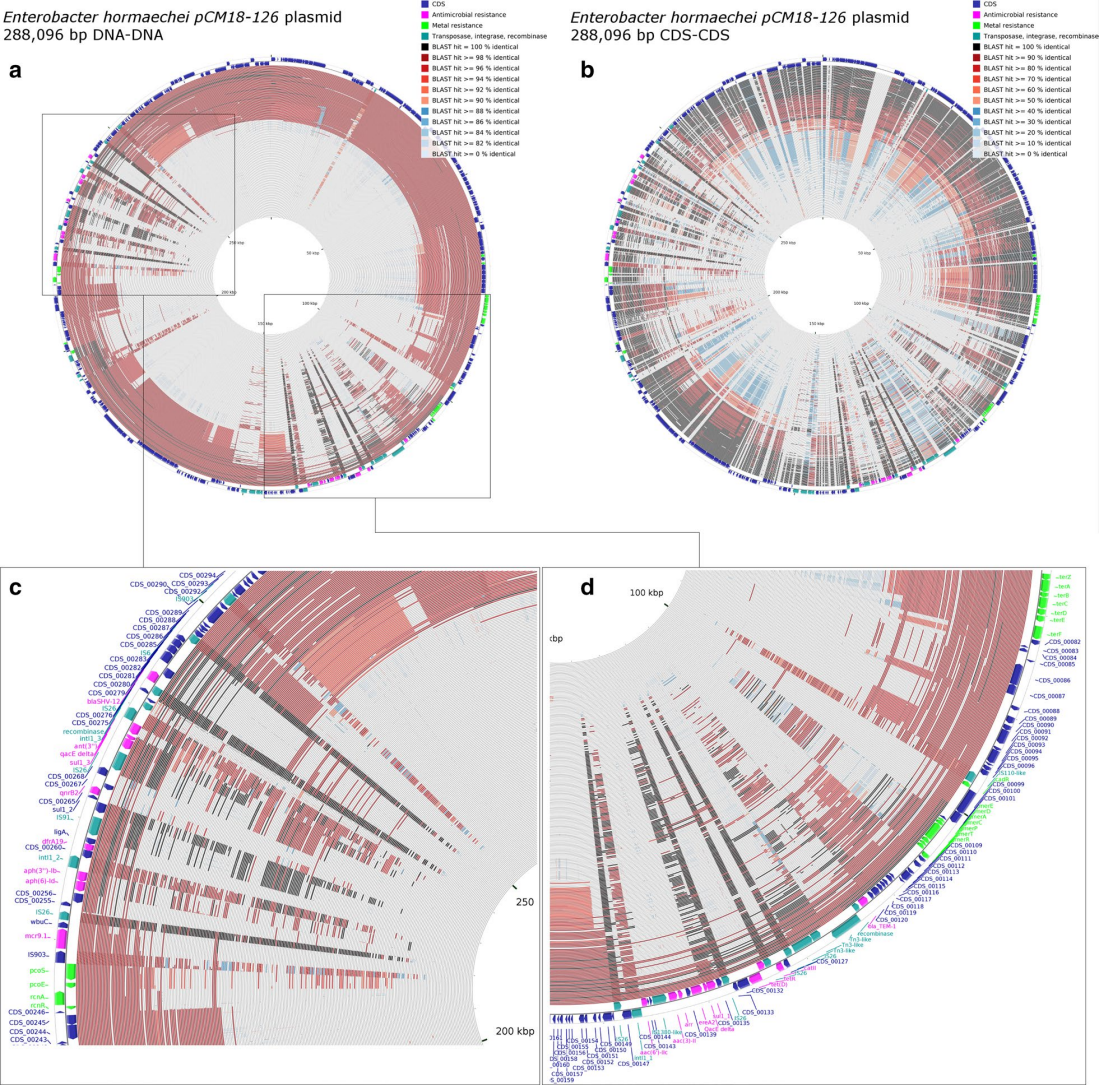
Comparative analysis of pCM18-216 against 269 complete plasmid sequences from *Enterobacteriaceae*. Outer rim represents the pCM18-216 map; ARGs are indicated in pink, metal resistance genes in green, transposable elements and integrases in teal. Each inner rim represents an individual plasmid sequence. **a** DNA-DNA alignments; **b** CDS-CDS alignments; **c** close-up view of ARG locus2; **d** close-up view of ARG locus1

Since the ARG loci-1 and -2 shared several genetic components with other multidrug resistance plasmids, the pCM18-216 sequence was compared to a set of 269 large plasmids of various incompatibility groups from *Enterobacteriaceae* (Additional file 1: Table S2). BLASTN DNA-DNA alignments showed that over 100 of these plasmids shared most of their sequence with pCM18-216 (Fig. 4a). However, BLASTP analysis of pCM18-216 CDS products indicated that some sequences were shared with only a smaller subset of replicons (Fig. 4b); for the most part these genes corresponded to ARG-carrying and mercury resistance loci of the plasmid (Fig. 4c, d). All 98 IncHI2 plasmids present in the dataset displayed overall sequence similarity with pCM18-216, but only 22 and 8 plasmids possessed a *bla*SHV-12 and *qnrB*2 gene, respectively.

A comparative analysis of IncH12 plasmids carrying *qnrB*2 and displaying high levels of similarity with pCM18-216 (Table 6) showed that they all shared a common backbone with a number of sequence re-arrangements and inversions (Additional file 4: Fig. S3). The pEC-IMPQ sequence was the most closely related to pCM18-216 with 99.94% sequence similarity, and carried an IS26-flanked composite transposon containing *bla*SHV-12 and a class 1 complex integron containing ISCR1 elements and *qnr*B2, but these components were located distantly on the replicon. Similarly, the plasmid p34977 from *Enterobacter hormaechei* subsp. *steigerwaltii* (CP_012170.1) possessed an IS26-*bla*SHV-12 transposon located 21 kbp away from the class 1 complex integron above described. By contrast, in pCM18-216 ARG locus-2, the IS26-*bla*SHV-12 transposon was immediately adjacent to the complex class 1 integron (Fig. 3b).

**Table 6:** IncH12 plasmids carrying *qnr*B2 selected for comparative analysis

Plasmid	NCBI ID	Assigned taxon	Host	Country	Year
pEC-IMPQ	NC_012556.1	Enterobacter cloacae	Human	Taiwan	2009
p34977-263	NZ_CP012170.1	*Enterobacter hormaechei* subspecies steigerwaltii	Human	USA	2015
p09-036813-1A_261	NZ_CP016526.1	Salmonella enterica subspecies enterica serovar Heidelberg	Horse	Canada	2016
pIMP4-SEM1	KX810825.1	Salmonella enterica subspecies enterica serovar Typhimurium	Cat	Australia	2016
pMS7884A	NZ_CP022533.1	*Enterobacter hormaechei*	Human	Australia	2017
Plasmid “unnamed-4”	NZ_CP029717.1	Enterobacter cloacae	-	USA	2018
pGMI14-002_1	NZ_CP028197.1	Salmonella enterica subspecies enterica serovar Concord	-	Czech Republic	2018

**Fig. 5:**
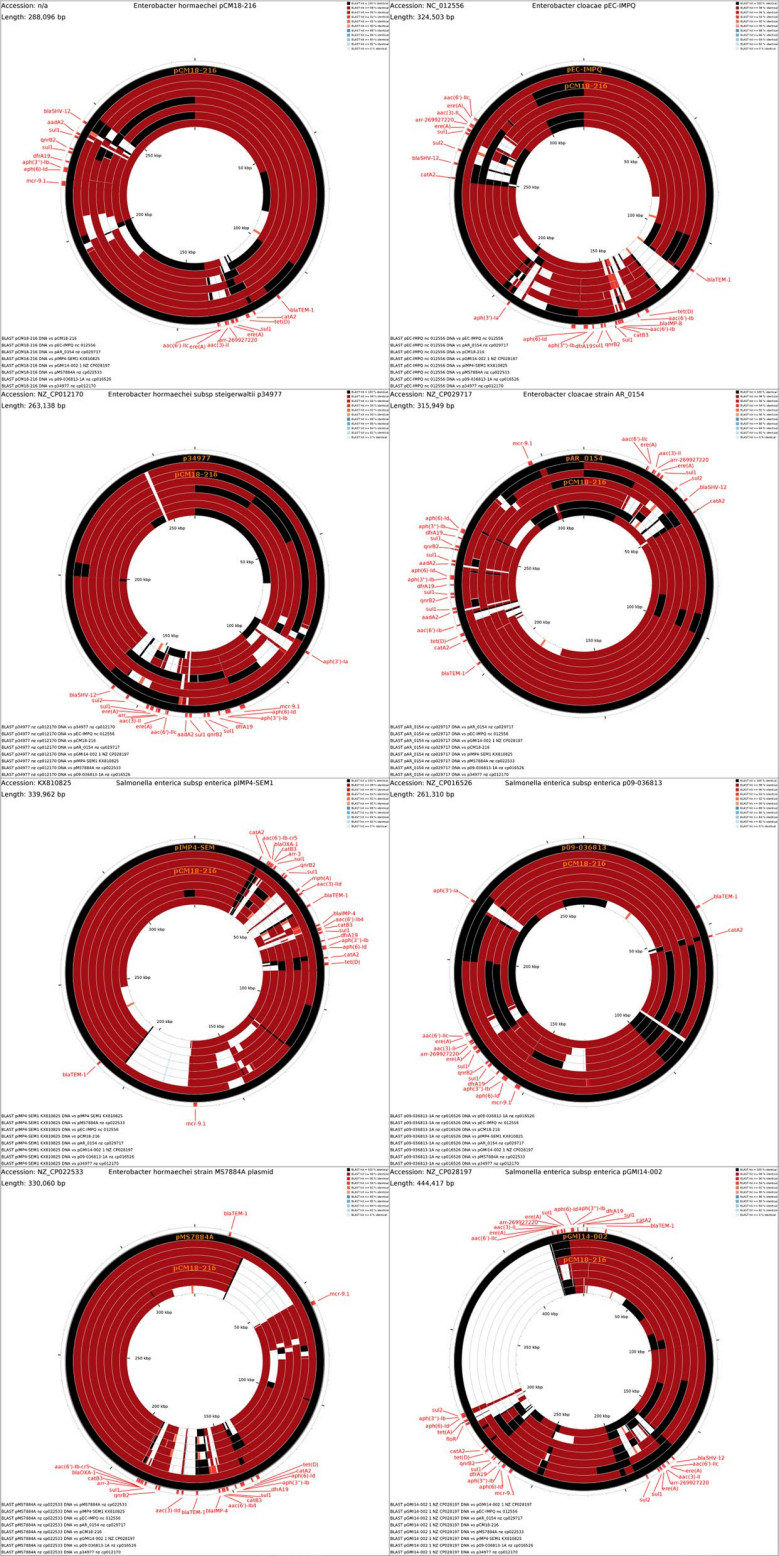
Systematic comparative alignments of pCM18-216 and *qnrB*2-carrying incH12 plasmids from *Enterobacteriaceae* (see Table 6 for details). Each panel represents a query sequence plasmid (black outer circle) and the seven subject sequences (inner circles) arranged by decreasing order of similarity with the query. Positions of antimicrobial resistance genes in each query sequence are indicated in red

Systematic alignments of these plasmids with the CGView Comparison tool confirmed that ARGs-carrrying regions are associated with most of the gene diversity within the subset (Fig. 5). While all plasmids except one carried an ESBL gene (*bla*SHV-12 or *bla*OXA1), only 3 plasmids (namely pEC-IMPQ, pIMP4-SEM1 and pMS7884A) also encoded metallo beta lactamases (*bla*IMP-4 or *bla*IMP-8) conferring resistance to carbapenems.

## Discussion

The veterinary hospital investigated in this study has been using a registered commercial disinfectant containing benzalkonium chloride and biguanide hydrochloride for regular decontamination procedures. This type of product is widely used in animal care premises as it is considered efficacious against common veterinary pathogens as well as being safe for pets and staff. For cleaning and disinfection of sinks, the Standard Operating Procedure (SOP) enforced in the hospital is performed in two steps. First, a detergent or scrubbing agent is used to remove most organic material, followed by a thorough rinsing with water. Then, the disinfectant is applied liberally and allowed to dry, ensuring a minimum 10 min contact time, as per the manufacturer instructions. Hospital staff members are supervised and trained by the Hospital Infection Control Officer (ICO) to ensure compliance with the SOP. The repeated isolation of *Enterobacter* in the hospital ICU exemplifies the capacity of some micro-organisms to persist in health care premises despite normal disinfection attempts. The plasmid-encoded efflux pump *qacE* delta1 may have played a role in conferring partial resistance against the quaternary ammonium compound present in the disinfectant. However we cannot rule out that the other factors, such as the presence of grooves or hard-to-reach parts in the sink structure, may have initially interfered with the correct application of the product. Here, the hospital ICO played a crucial role to ensure that proper decontamination protocols were followed, including the manufacturer’s recommendations for dilution, temperature and contact time of the disinfectant. The *Enterobacter* strain was not detected from swabs collected after a third round of decontamination of the sink, suggesting that the correct measures were eventually applied with success. Benzalkonium chloride is still used in the veterinary hospital. Routine environmental surveillance of the premises has not indicated the presence of intractable infectious agents, when the disinfectant is applied correctly.

Although two biochemical identification kits classified the *Enterobacter* isolates as *E. cloacae*, the absence of lactose fermentation was atypical for this species [36], as 93% of *E. cloacae* strains but only 9% of *E. hormaechei* strains appear lactose positive on MacConkey plates after 48 h [37]. Within the *E. cloacae* complex, accurate species identification by MALDI-TOF can be difficult, prompting for DNA sequencing to resolve taxonomic ambiguities [38]. The various genome analysis methods used in our study (Ribosomal Multilocus Sequence Typing, Average Nucleotide Identity and pylogenetic tree construction) identified the sink isolates as *E. hormaechei*. These results illustrate the current limitations of identification kits and databases for the correct classification of species in the *E. cloacae* complex.

All chromosomal ARGs were components of multidrug efflux pumps, except for *bacA*, which confers resistance to bacitracin by target alteration [39], whereas the conjugative plasmid pCM18-216 encoded specific resistance mechanisms against important antimicrobials, such as fluoroquinolones and cephalosporins. Although no ECOFF value is currently available for *E. hormaechei* against enrofloxacin, the MIC of 1 ug/mL observed with this antimicrobial was well above the ECOFF value of 0.125 ug/mL reported by EUCAST for *E. coli*, suggesting the presence of an acquired (albeit modest) resistance to the drug, likely due to the *qnr*B2 gene. However, the *E. coli* transconjugants carrying pCM18-216 were susceptible to fluoroquinolones. The reason for this is unclear, but the impact of a *qnr*B2 resistance on therapeutic outcomes in animals infected by *E. hormaechei* or other nosocomial agents carrying pCM18-216 cannot be dismissed. The pCM18-216 carried the *mcr*-9.1 gene, encoding a newly described phosphoethanolamine transferase which can confer an inducible resistance to colistin upon exposure to sub-inhibitory concentrations of the drug [40]. The sink isolates appeared susceptible to colistin and polymyxin B based on conventional testing methods. Although preliminary attempts at inducing colistin resistance in CM18-216 and CM18-242–2 by sub-culturing the isolates in presence of the antibiotic in broth or solid media failed to demonstrate a reversible increase in MIC in our hands, this question deserves further scrutiny. In *E. coli*, the two component system encoded by *qseC* and *qseB* is proposed to regulate the expression of polymixin/colistin resistance [41]. These genes are localised next to *mcr*-9.1 and IS903 in some *E. coli* and *E. hormaechei* plasmids [40]. While *qseC* and *qseB* were not carried pCM18-216, homologous sequences were found on the isolate chromosome, between nt positions 691981 and 693986. It is unclear whether *E. hormaechei* CM18-216 can display colistin resistance under certain inducing conditions, which remain to be defined, but as this antimicrobial is a last resort, high importance drug for humans, the presence of an organism carrying *mcr*-9.1 in a veterinary ICU is concerning.

The co-selection of resistant organisms and propagation of resistance genes in veterinary hospital environments has been explored recently in our group, with a particular focus on the ICU [42]. The phenotypic characterization of metal resistances in the sink isolates was beyond the scope of this study, but it is worth noticing that pCM18-216 carried tellurium resistance gene clusters typically found in IncH12 plasmids [43] and heavy metal resistance genes organized similarly to other plasmids and transposons of Gram negative bacteria [44, 45]. The ESBL production was putatively attributed to the plasmidic gene *bla*SHV-12; the association of ESBL-encoding and metal resistance genes has been recently reported in *E. hormaechei* [46]. Topical preparations containing silver and fluoroquinolones are commercially available in Australia for the treatment of ear infections in companion animals, raising questions about the risks associated with the accumulation of heavy metals and antimicrobials residues in veterinary premises. The temperature requirements observed in mating experiments between CM18-216 and *E. coli* are also found in the conjugative transfer of IncHI plasmids, which occurs only within a 22–28 °C range [34, 47]. This suggests that pCM18-216 can transfer from *E. hormaechei* to other bacteria and disseminate heavy metal and multidrug resistances, including ESBLs, in the hospital normal environmental conditions.

The isolates also carried several mobile genetic elements. The presence of an additional chromosomal prophage in one of the four isolates indicates that the *Enterobacter* population colonizing the ICU sink may have acquired or rearranged mobile genetic elements over time. Several transposases and integrases were also found in the plasmid sequence, with important consequences for the physical organisation and potential co-transfer of ARGs. In Australia, ISCR1 have been described in IncL/M plasmids and IS26-associated class 1 integrons carrying *qnrB2* [48]. The ISCR1 elements are involved in rolling-circle transposition to form complex class 1 integrons [49]. IS26 mediated genetic re-arrangements are also well documented [50–52], particularly for their role in dissemination of antimicrobial resistance genes. The accumulation of antimicrobial resistance genes in genetic loci flanked by IS26 elements was more pronounced in pCM18-216 compared to other plasmids. No other sequence in the Genbank nucleotide database possessed a complete colinearity with the pCM18-216 full ARG locus-2, suggesting that this structure was created by intra-plasmidic sequence relocation. Because of the physical proximity of these ARGs and the presence of two bordering IS903 copies, the ARG locus-2 of pCM18-216 has the potential to facilitate the simultaneous horizontal gene transfer of the ESBL gene *bla*SHV-12 and the fluoroquinolone resistance gene *qnr*B2, along with other antimicrobial resistance genes, through a single transposition event. In Australia, *bla*IMP-4 genes have been associated with IncHI2 plasmids carried by *E. hormaechei* with various MLST profiles, but only two ST110 isolates [53]. These antimicrobials are considered of very high importance and their use in companion animals is not generally recommended (https://vetantibiotics.fvas.unimelb.edu.au/). Although the isolates CM18-216 and CM18-242 were susceptible to carbapenems and did not carry *bla*IMP sequences on their plasmids, the presence of the same mobile genetic elements found on *bla*IMP plasmids and pCM18-216 opens the question whether the organism is able to acquire such resistance. This underlines the importance of early detection of multidrug resistant organisms and decontamination to control the risks of dissemination of resistance within the hospital.

## Conclusions

The presence in the veterinary hospital ICU of an ESBL, as well as fluoroquinolone and putative colistin resistance genes within an IS26 transposon in a conjugative plasmid for nearly one month underlines the risk of horizontal dissemination of ARGs into other bacterial species and nosocomial infections with reduced possibilities of treatment. This was concerning, even though the *Enterobacter* host was not phenotypically resistant to colistin and presented only intermediate MICs levels against ciprofloxacin. Repeated rounds of disinfection of the sink pipes were implemented until the organism could no longer be detected by environmental sampling. Based on these results, routine environmental surveillance programs incorporating the rapid detection of organisms capable of ESBL production and resistance to fluoroquinolones, colistin and carbapenems, should be considered in large veterinary hospitals.

## Supplementary information

**Additional file 1: Table S1**. Sequencing read statistics after quality filtering. Table S2. Details on the genomes used to construct the phylogenetic tree.

**Additional file 2: Figure S1**. Chromosomal map of the E. hormaechei isolate CM18-216. From outer to inner circles: 1, nucleotide positions; 2 and 3, CDSs (grey); 4, tRNA (green); 5, predicted genomic islands and prophages (red); 6, pco/sil copper/silver resistance(brown-green), transposases (purple), salmochelin synthesis and uptake (blue); 7, GC% plot; 8, GC skew plot. Inset: detailed map of the pco/sil resistance locus.

**Additional file 3: Figure S2**. Mauve alignment of the chromosome sequences of the four Enterobacter strains isolated from the ICU sink over approximately one month. Local blocks of colinearity are labelled with different colors. Predicted coding sequences are indicated underneath each genome. CM18-216 and CM18-242-2 (two top rows) were obtained from hybrid assemblies of Illumina and Nanopore reads; CM18-269-1 and CM18-269-2 (two bottom rows) were assembled from nanopore reads only. Position of a putative phage in CM18-269-2 is indicated by a red box.

**Additional file 4: Figure S3**. Alignment of pCM18-216 with IncH12 plasmids carrying qnrB2. Horizontal black lines indicate the lengths of the plasmid sequences. Dark blue horizontal bars on the top (forward strand) and the bottom (reverse strand) of the black lines indicate areas of sequence homology. Vertical bars connecting the horizontal lines show areas of sequence homology.

## Data Availability

The datasets generated and analysed during the current study have been deposited in the Genbank repository under the following entries: BioProject PRJNA613546; BioSample SAMN14409014: CP05031 (chromosome CM18-216), CP050312 (plasmid pCM18-216); BioSample SAMN14449833: CP050506 (chromosome CM18-242-2), CP050507 (plasmid pCM18-242-2).

## Abbreviations

ICU: Intensive care units
ESBL: Extended spectrum beta-lactamase
MIC: Minimum inhibitory concentration
ARG: Antimicrobial resistance gene
