# Supplementary material for: Colonization of a hand washing sink in a veterinary hospital by an Enterobacter hormaechei strain carrying multiple resistances to high importance antimicrobials
Source: Antimicrob Resist Infect Control. 2020 Oct 21;9:163. doi: 10.1186/s13756-020-00828-0 (PMC7580002; doi:10.1186/s13756-020-00828-0)
Supplement: Supplementary file 1 — Additional file 1: Table S1. Sequencing read statistics after quality filtering. Table S2. Details on the genomes used to construct the phylogenetic tree. [file 13756_2020_828_MOESM1_ESM.docx]

Table S1. Sequencing read statistics after quality filtering

| Isolate | Nanopore | | | | Illumina | |
| --- | --- | --- | --- | --- | --- | --- |
|  | No. reads | Total sequence (Mbp) | Mean read length (kbp) | N50  (kbp) | No. reads | Read length (bp) |
| CM18-216 | 8,519 | 129 | 15 | 21 | 2,061,010 | 110-125 |
| CM18-242-2 | 17,872 | 271 | 15 | 21 | 1,759,821 | 115-125 |
| CM18_269_1 | 20,209 | 389 | 19 | 25 | NA | NA |
| CM18_269_2 | 23,373 | 398 | 17 | 22 | NA | NA |

Table S2. Details on the genomes used to construct the phylogenetic tree.

| Refseq Assembly | Species | Strain | Host | Source | Isolation Location | Isolation Date |
| --- | --- | --- | --- | --- | --- | --- |
| GCF_000016325.1_ASM1632v1 | *Enterobacter* sp. | 638 | n/a | n/a | n/a | n/a |
| GCF_000017665.1_ASM1766v1 | *Cronobacter sakazakii* | ATCC_BAA-894 | n/a | n/a | n/a | n/a |
| GCF_000025565.1_ASM2556v1 | *Enterobacter cloacae* subsp. *cloacae* | ATCC_13047 | n/a | n/a | n/a | n/a |
| GCF_000224675.1_ASM22467v1 | *Enterobacter soli* | LF7a | n/a | n/a | n/a | n/a |
| GCF_000235765.1_ASM23576v3 | *Enterobacter cloacae* subsp. *dissolvens* | SDM | n/a | n/a | n/a | n/a |
| GCF_000239975.1_ASM23997v1 | *Enterobacter ludwigii* | EcWSU1 | n/a | n/a | n/a | n/a |
| GCF_000286275.1_ASM28627v1 | *Enterobacter kobei* | ENHKU01 | n/a | n/a | n/a | n/a |
| GCF_000410515.1_ASM41051v1 | *Enterobacter* sp. | R4-368 | n/a | n/a | n/a | n/a |
| GCF_000512375.1_ASM51237v1 | *Enterobacter ludwigii* | P101 | n/a | n/a | n/a | n/a |
| GCF_000632395.1_ASM63239v1 | *Enterobacter asburiae* | L1 | n/a | lettuce | Malaysia | 03-Apr-2013 |
| GCF_000724505.1_ASM72450v1 | *Enterobacter cloacae* | ECNIH2 | n/a | sink drain | USA | 2012 |
| GCF_000750225.1_ASM75022v1 | *Enterobacter hormaechei* subsp. *hoffmannii* | ECNIH3 | Homo sapiens | tracheal aspirate | USA | 2011 |
| GCF_000750275.1_ASM75027v1 | *Enterobacter hormaechei* subsp. *hoffmannii* | ECR091 | Homo sapiens | urine | USA | 2012 |
| GCF_000770155.1_ASM77015v1 | *Enterobacter cloacae* | GGT036 | n/a | n/a | South Korea | n/a |
| GCF_000783675.2_ASM78367v2 | *Enterobacter cloacae complex* sp. | FDAARGOS_77 | Homo sapiens | Rectal Swab | USA | 07-Sep-2013 |
| GCF_000784865.1_ASM78486v1 | *Enterobacter cloacae* | ECNIH4 | n/a | sink drain | USA | 2012 |
| GCF_000784905.1_ASM78490v1 | *Enterobacter cloacae* | ECNIH5 | n/a | sink drain | USA | 2011 |
| GCF_000801755.2_ASM80175v2 | *Enterobacter* sp. | E20 | Oryza sativa | glyphosate polluted soil | China Zhejiang | Oct-2010 |
| GCF_000807405.2_ASM80740v4 | *Enterobacter hormaechei* subsp. *oharae* | 34978 | Homo sapiens | bodily fluid | USA | 2011 |
| GCF_000807415.2_ASM80741v4 | *Enterobacter roggenkampii* | 35734 | Homo sapiens | Excreted bodily substance | USA | 2010 |
| GCF_000807425.2_ASM80742v4 | *Enterobacter hormaechei* subsp. *steigerwaltii* | 34998 | Homo sapiens | bodily fluid | USA | 2011 |
| GCF_000814125.3_ASM81412v3 | *Enterobacter hormaechei* subsp. *steigerwaltii* | 34977 | Homo sapiens | bodily fluid | USA | 2009 |
| GCF_000814205.1_ASM81420v1 | *Enterobacter hormaechei* subsp. *hormaechei* | 34983 | Homo sapiens | bodily fluid | USA | 2010 |
| GCF_000814225.1_ASM81422v1 | *Enterobacter hormaechei* subsp. *xiangfangensis* | 34399 | Homo sapiens | Excreted bodily substance | USA | 2011 |
| GCF_001022015.1_ASM102201v1 | *Enterobacter hormaechei* | CAV1311 | Homo sapiens | Urine Genitourinary | USA Virginia | 2011-01 |
| GCF_001022055.1_ASM102205v1 | *Enterobacter hormaechei* | CAV1668 | Homo sapiens | Perirectal | USA Virginia | 2012-08 |
| GCF_001022075.1_ASM102207v1 | *Enterobacter hormaechei* | CAV1411 | Homo sapiens | Respiratory | USA Virginia | 2011-06 |
| GCF_001022095.1_ASM102209v1 | *Enterobacter asburiae* | CAV1043 | Homo sapiens | n/a | USA Virginia | 2008-03 |
| GCF_001022255.1_ASM102225v1 | *Enterobacter hormaechei* | CAV1669 | Homo sapiens | Perirectal | USA Virginia | 2012-08 |
| GCF_001029645.1_ASM102964v1 | *Enterobacter ludwigii* | UW5 | n/a | soil | Canada Waterloo | 29-Sep-1994 |
| GCF_001521715.1_ASM152171v1 | *Enterobacter asburiae* | ATCC_35953 | Homo sapiens | physical | n/a | 2014-12-01 |
| GCF_001617645.1_ASM161764v1 | *Enterobacter asburiae* | ENIPBJ-CG1 | Homo sapiens | n/a | China Beijing | 2014-07-03 |
| GCF_001623605.1_ASM162360v1 | *Enterobacter* sp. | ODB01 | n/a | crude oil contaminated soil | China | 2014-09-01 |
| GCF_001719105.1_ASM171910v1 | *Enterobacter* sp. | HK169 | n/a | Tomato roots | South Korea Daejeon | 2015 |
| GCF_001729705.1_ASM172970v1 | *Enterobacter hormaechei* subsp. *oharae* | DSM_16687 | Homo sapiens | n/a | Germany | n/a |
| GCF_001729725.1_ASM172972v1 | *Enterobacter hormaechei* subsp. *steigerwaltii* | DSM_16691 | Homo sapiens | wound | Belgium | n/a |
| GCF_001729745.1_ASM172974v1 | *Enterobacter hormaechei* subsp. *hoffmannii* | DSM_14563 | Homo sapiens | n/a | Germany | n/a |
| GCF_001729765.1_ASM172976v1 | *Enterobacter kobei* | DSM_13645 | Homo sapiens | blood | Japan | n/a |
| GCF_001729785.1_ASM172978v1 | *Enterobacter hormaechei* subsp. *xiangfangensis* | LMG27195 | n/a | Chinese traditional sourdough | China Heilongjiang province | n/a |
| GCF_001729805.1_ASM172980v1 | *Enterobacter roggenkampii* | DSM_16690 | Homo sapiens | n/a | Germany | n/a |
| GCF_001750725.1_ASM175072v1 | *Enterobacter ludwigii* | EN-119 | Homo sapiens | n/a | n/a | n/a |
| GCF_001874505.1_ASM187450v1 | *Enterobacter hormaechei* | CAV1176 | Homo sapiens | Perirectal | USA Virginia | 2010-05 |
| GCF_001888805.2_ASM188880v2 | *Enterobacter* sp. | SA187 | n/a | roots of desert plants | Saudi Arabia Jizan | 01-Mar-2014 |
| GCF_001922365.1_ASM192236v1 | *Enterobacter cloacae* | AR_0002 | n/a | n/a | n/a | n/a |
| GCF_001984825.2_ASM198482v2 | *Enterobacter chengduensis* | WCHECl-C4 | Homo sapiens | blood | China Chengdu Sichuan | 2015-02-01 |
| GCF_002007805.1_ASM200780v1 | *Enterobacter roggenkampii* | R11 | n/a | sewage water | China Shandong | 2016-12-17 |
| GCF_002025685.1_ASM202568v1 | *Enterobacter ludwigii* | AA4 | Zea mays | root | USA Boston | Dec-2012 |
| GCF_002055735.1_ASM205573v1 | *Enterobacter cloacae* | AR_0065 | n/a | n/a | n/a | n/a |
| GCF_002192355.1_ASM219235v1 | *Enterobacter cloacae* | AR_0163 | n/a | n/a | n/a | n/a |
| GCF_002192395.1_ASM219239v1 | *Enterobacter cloacae* | AR_0053 | n/a | n/a | n/a | n/a |
| GCF_002197345.1_ASM219734v1 | *Enterobacter cloacae* | A1137 | Homo sapiens | blood | n/a | n/a |
| GCF_002201815.1_ASM220181v1 | *Enterobacter cloacae* | AR_0050 | n/a | n/a | n/a | n/a |
| GCF_002204775.1_ASM220477v1 | *Enterobacter cloacae* | AR_0136 | n/a | n/a | n/a | n/a |
| GCF_002208095.1_ASM220809v1 | *Enterobacter cloacae complex* sp. | ECNIH7 | n/a | n/a | USA | 2014 |
| GCF_002211685.1_ASM221168v1 | *Enterobacter roggenkampii* | 704SK10 | n/a | wastewater | Switzerland Basel | Dec-2015 |
| GCF_002237465.1_ASM223746v1 | *Enterobacter hormaechei* | MS7884A | Homo sapiens | endotracheal tube | Australia | 15-Jun-2015 |
| GCF_002303275.1_ASM230327v1 | *Enterobacter cloacae* | M12X01451 | Homo sapiens | Stool | n/a | n/a |
| GCF_002787395.1_ASM278739v1 | *Enterobacter* sp. | CRENT-193 | Homo sapiens | wound | South Korea Seoul | 2013 |
| GCF_002850575.1_ASM285057v1 | *Enterobacter cancerogenus* | CR-Eb1 | Galleria mellonella | gut 3 4th instar larva | South Korea Daejeon | 2015-09 |
| GCF_002863825.1_ASM286382v1 | *Enterobacter* sp. | Crenshaw | Rhizoctonia solani | brown patch in grass | USA Kansas | 12-Dec-2016 |
| GCF_002947755.1_ASM294775v1 | *Enterobacter cloacae* | AR_0060 | n/a | n/a | n/a | n/a |
| GCF_002954165.1_ASM295416v1 | *Enterobacter cloacae* | AR_0072 | n/a | n/a | n/a | n/a |
| GCF_002968455.1_ASM296845v1 | *Enterobacter hormaechei* subsp. *hoffmannii* | AR_0365 | n/a | n/a | n/a | n/a |
| GCF_002982195.1_ASM298219v1 | *Enterobacter cloacae* | PIMB10EC27 | Homo sapiens | urine | Viet Nam | 2010 |
| GCF_003010695.1_ASM301069v1 | *Enterobacter cloacae complex* sp. | FDA-CDC-AR_0132 | n/a | n/a | n/a | n/a |
| GCF_003031445.1_ASM303144v1 | *Enterobacter cloacae* | 109 | Homo sapiens | trachael aspirate | USA Boston | 2015 |
| GCF_003031755.1_ASM303175v1 | *Enterobacter cloacae* | 174 | Homo sapiens | blood | USA Boston | 2015 |
| GCF_003051945.2_ASM305194v2 | *Enterobacter hormaechei* | SCEH020042 | Homo sapiens | n/a | China Panzhihua Sichuan | 2016-10-10 |
| GCF_003053755.1_ASM305375v1 | *Enterobacter cloacae* | AR_0093 | n/a | n/a | n/a | n/a |
| GCF_003071645.1_ASM307164v1 | *Enterobacter cloacae complex* sp. | FDA-CDC-AR_0164 | n/a | n/a | n/a | n/a |
| GCF_003073995.1_ASM307399v1 | *Enterobacter hormaechei* | AR432 | n/a | n/a | n/a | n/a |
| GCF_003186415.1_ASM318641v1 | *Enterobacter hormaechei* | 234 | Homo sapiens | wound | USA Boston | 2016 |
| GCF_003186565.1_ASM318656v1 | *Enterobacter hormaechei* | 388 | n/a | n/a | USA Boston | 2017 |
| GCF_003204095.1_ASM320409v1 | *Enterobacter cloacae* | AR_0154 | n/a | n/a | n/a | n/a |
| GCF_003254805.1_ASM325480v1 | *Enterobacter hormaechei* subsp. *xiangfangensis* | Pb204 | n/a | acid mine decant and tailings from | South Africa West Rand Gauteng | Jan-2014 |
| GCF_003264955.1_ASM326495v1 | *Enterobacter hormaechei* | 20710 | Homo sapiens | sptum | China Shandong | 2011-08 |
| GCF_003288475.1_ASM328847v1 | *Enterobacter hormaechei* | AR_038 | n/a | n/a | n/a | n/a |
| GCF_003382725.1_ASM338272v1 | *Enterobacter hormaechei* subsp. *xiangfangensis* | OSUVMCKPC4-2 | canine | n/a | USA Ohio Columbus | 2016-07-27 |
| GCF_003408555.1_ASM340855v1 | *Enterobacter hormaechei* | 2013_1a | Homo sapiens | Rectal Swab | n/a | 2013 |
| GCF_003408575.1_ASM340857v1 | *Enterobacter hormaechei* | N1 | Homo sapiens | Rectal Swab | n/a | 2015 |
| GCF_003408595.1_ASM340859v1 | *Enterobacter hormaechei* | A1 | Homo sapiens | Rectal Swab | n/a | 2015 |
| GCF_003428425.1_ASM342842v1 | *Enterobacter hormaechei* | WCHEH020038 | Homo sapiens | n/a | China Sichuan Chengdu | 2016-11-02 |
| GCF_003444755.1_ASM344475v1 | *Enterobacter hormaechei* | FRM | n/a | high concentration of fluoride | China | 2008-10-30 |
| GCF_003586025.1_ASM358602v1 | *Enterobacter hormaechei* subsp. *xiangfangensis* | OSUKPC4_L | Canis lupus familiaris | Bite Wound | USA Ohio Columbus | 09-Sep-2016 |
| GCF_003660125.1_ASM366012v1 | *Enterobacter hormaechei* | C15117 | n/a | Burns unit surveillance | Australia Sydney | 2007 |
| GCF_003665375.1_ASM366537v1 | *Enterobacter kobei* | WCHEK045523 | Homo sapiens | n/a | China Sichuan Chengdu | 2017 |
| GCF_003719615.1_ASM371961v1 | *Enterobacter cloacae* | E3442 | Penaeus vannamei | n/a | Netherlands | Mar-2017 |
| GCF_003812145.1_ASM381214v1 | *Enterobacter roggenkampii* | FDAARGOS_523 | Homo sapiens | Rectal Swab | n/a | 28-Dec-2015 |
| GCF_003940765.1_ASM394076v1 | *Enterobacter asburiae* | CAV1043 | n/a | water | USA | 18-Jun-2018 |
| GCF_003964795.2_ASM396479v2 | *Enterobacter hormaechei* subsp. *xiangfangensis* | WCHEX045001 | Homo sapiens | blood | China Chengdu Sichuan | 2018-01 |
| GCF_003965345.2_ASM396534v2 | *Enterobacter hormaechei* | WCHEH090011 | Homo sapiens | n/a | China Chengdu Sichuan | 2017 |
| GCF_004006055.1_ASM400605v1 | *Enterobacter* sp. | N18-03635 | Homo sapiens | Rectal Swab | Canada | n/a |
| GCF_004118875.1_ASM411887v1 | *Enterobacter hormaechei* | S11_16 | Homo sapiens | n/a | United Kingdom | 2016 |
| GCF_004138605.1_ASM413860v1 | *Enterobacter roggenkampii* | ECY546 | Homo sapiens | n/a | China Wenzhou | 2008-10-30 |
| GCF_004151605.1_ASM415160v1 | *Enterobacter cloacae* | EN3600 | Homo sapiens | blood | China Anhui | May-2015 |
| GCF_004193715.1_ASM419371v1 | *Enterobacter cloacae* | CZ-1 | n/a | paddy soil | China Zhenzhou | 14-Jul-2016 |
| GCF_004355165.1_ASM435516v1 | *Enterobacter cloacae complex* sp. | N13-01531 | Homo sapiens | n/a | Canada Alberta | 2013 |
| GCF_004684365.1_ASM468436v1 | *Enterobacter roggenkampii* | BP10374 | Homo sapiens | blood | India | 2018 |
| GCF_004804375.1_ASM480437v1 | *Enterobacter bugandensis* | 220 | Homo sapiens | throat swab | Germany Hessen | 15-Jan-2019 |
| GCF_004804395.1_ASM480439v1 | *Enterobacter bugandensis* | 1367 | Homo sapiens | blood | Germany North Rhine Westphalia | 01-Jan-2011 |
| GCF_005518115.1_ASM551811v1 | *Enterobacter ludwigii* | JP6 | Tobacco | rhizosphere soil | China Hunan Province | 2011-05-01 |
| GCF_005848825.1_ASM584882v1 | *Enterobacter ludwigii* | JP9 | n/a | Tobacco rhizosphere soil | China Hunan Province | 2011-05-01 |
| GCF_005890075.1_ASM589007v1 | *Enterobacter ludwigii* | I42 | n/a | Lycium barbarum rhizosphere soil | China Ningxia Hui Autonomous Region | 2015-09-01 |
| GCF_006228165.1_ASM622816v1 | *Enterobacter cloacae* | NH77 | Homo sapiens | n/a | Thailand Chiang Mai | Jan-2018 |
| GCF_006385655.1_ASM638565v1 | *Enterobacter hormaechei* | C126 | n/a | urine | India | 2016 |
| GCF_006385915.1_ASM638591v1 | *Enterobacter ludwigii* | I140 | n/a | soil | China Ningxia Hui Autonomous Region | 2011-05-01 |
| GCF_007035645.1_ASM703564v1 | *Enterobacter asburiae* | 1808-013 | Homo sapines | urine | Japan Osaka | 2018-08 |
| GCF_007035805.1_ASM703580v1 | *Enterobacter asburiae* | 17Nkhm-UP2 | n/a | river water | Japan Osaka | 2017-09 |
| GCF_007035975.1_ASM703597v1 | *Enterobacter* sp. | 18A13 | n/a | river water | Japan Osaka | 2018-08 |
| GCF_007556795.1_ASM755679v1 | *Enterobacter hormaechei* subsp. *steigerwaltii* | ME-1 | n/a | n/a | n/a | 2018 |
| GCF_008123985.1_ASM812398v1 | *Enterobacter hormaechei* | PG20180056 | n/a | mouse gut | n/a | 18-May-2018 |
| GCF_008124025.1_ASM812402v1 | *Enterobacter hormaechei* | PG20180049 | n/a | mouse gut | n/a | 18-May-2018 |
| GCF_008271405.1_ASM827140v1 | *Enterobacter* sp. | LU1 | goat | rumen content | Poland Lubelskie Voivodeship | 2010-2011 |
| GCF_008365235.1_ASM836523v1 | *Enterobacter kobei* | EB_P8_L5_01.19 | Homo sapiens | screening swab | United Kingdom London | 2019-01 |
| GCF_008505035.1_ASM850503v1 | *Enterobacter hormaechei* | EB_P6_L3_02.19 | Homo sapiens | rectal screen | United Kingdom London | 2019-02-27 |
| GCF_008693905.1_ASM869390v1 | *Enterobacter hormaechei* | FDAARGOS_642 | Homo sapiens | clinical isolate | USA KY | n/a |
| GCF_008931325.1_ASM893132v1 | *Enterobacter hormaechei* | C44 | Homo sapiens | clinical sample | Australia Sydney | 15-Apr-2013 |
| GCF_008931405.1_ASM893140v1 | *Enterobacter hormaechei* | C15 | Homo sapiens | clinical sample | Australia Sydney | 23-Feb-2009 |
| GCF_008931465.1_ASM893146v1 | *Enterobacter* sp. | E76 | n/a | Shower 3 | Australia Sydney | 07-May-2014 |
| GCF_008931525.1_ASM893152v1 | *Enterobacter hormaechei* | E5 | n/a | Shower 3 | Australia Sydney | 13-Jun-2012 |
| GCF_008931545.1_ASM893154v1 | *Enterobacter kobei* | C16 | Homo sapiens | clinical sample | Australia Sydney | 12-Mar-2009 |
| GCF_008931585.1_ASM893158v1 | *Enterobacter hormaechei* | C4 | Homo sapiens | clinical sample | Australia Sydney | 28-Jun-2007 |
| GCF_008931645.1_ASM893164v1 | *Enterobacter hormaechei* | C45 | Homo sapiens | clinical sample | Australia Sydney | 27-Apr-2013 |
| GCF_008931785.1_ASM893178v1 | *Enterobacter hormaechei* | EB_P9_L5_03.19 | Homo sapiens | Rectal Swab | United Kingdom London | 2019-03 |
| GCF_009036245.1_ASM903624v1 | *Enterobacter cloacae* | SGAir0282 | n/a | air | Singapore | 14-May-2015 |
| GCF_009176645.1_ASM917664v1 | *Enterobacter oligotrophica* | CCA6 | n/a | n/a | Japan Hiroshima Higashi Hiroshima | n/a |
| GCF_009184765.2_ASM918476v2 | *Enterobacter roggenkampii* | WCHER090065 | Homo sapiens | n/a | China Chengdu Sichuan | 2016-08 |
| GCF_009497055.1_ASM949705v1 | *Enterobacter hormaechei* | AUH-ENM30 | Homo sapiens | n/a | Lebanon | 2016 |
| GCF_009648915.1_ASM964891v1 | *Enterobacter cancerogenus* | MiY-F | n/a | Cilantro | USA MI | 2014-12-10 |
| GCF_009707405.1_ASM970740v1 | *Enterobacter cloacae* | CBG15936 | Homo sapiens | sputum | China Guangzhou | 29-Mar-2017 |
| GCF_009728975.1_ASM972897v1 | *Enterobacter hormaechei* | ECL69214 | Homo sapiens | urine sample | China Guangzhou | 04-Feb-2018 |
| GCF_009738085.1_ASM973808v1 | *Enterobacter hormaechei* | L51 | Homo sapiens | feces | China Zhejiang | 2016-04-08 |
| GCF_009755685.1_ASM975568v1 | *Enterobacter asburiae* | AEB30 | n/a | ginger | USA Albany California | Jul-2015 |
| GCF_009834325.1_ASM983432v1 | *Enterobacter hormaechei* subsp. *hoffmannii* | MYJARB-EH1 | Homo sapiens | n/a | USA | 2018 |
| GCF_009905155.1_ASM990515v1 | *Enterobacter hormaechei* | F2 | n/a | River | China Guangzhou Guangdong | 2015-06-01 |
| GCF_009930835.1_ASM993083v1 | *Enterobacter hormaechei* | BW | Homo sapiens | Blood and wound | USA University of Michigan University Hospital | 2013-08-24 |
| GCF_009930935.1_ASM993093v1 | *Enterobacter hormaechei* | BW | Homo sapiens | n/a | USA Ann Arbor Michigan | 2014-05-30 |
| GCF_900322715.1_C309 | *Enterobacter hormaechei* subsp. *steigerwaltii* | C309 | Homo sapiens | hospital | France | 2015 |
| GCF_900322725.1_C45 | *Enterobacter cloacae complex* sp. | C45 | Homo sapiens | hospital | France | 2014 |
| GCF_900324475.1_EB-247 | *Enterobacter bugandensis* | EB247 | Homo sapiens | blood | Tanzania | 2010-01-05 |
| GCF_900497145.1_ASM90049714v1 | *Enterobacter cloacae* | EC-TO80 | n/a | n/a | n/a | n/a |
| GCF_900635705.1_32407_E01 | *Enterobacter cloacae* | NCTC11571 | n/a | n/a | n/a | 1800_2017 |
