# Supplementary figures and images for: Colonization of a hand washing sink in a veterinary hospital by an Enterobacter hormaechei strain carrying multiple resistances to high importance antimicrobials
Source: Antimicrob Resist Infect Control. 2020 Oct 21;9:163. doi: 10.1186/s13756-020-00828-0 (PMC7580002; doi:10.1186/s13756-020-00828-0)

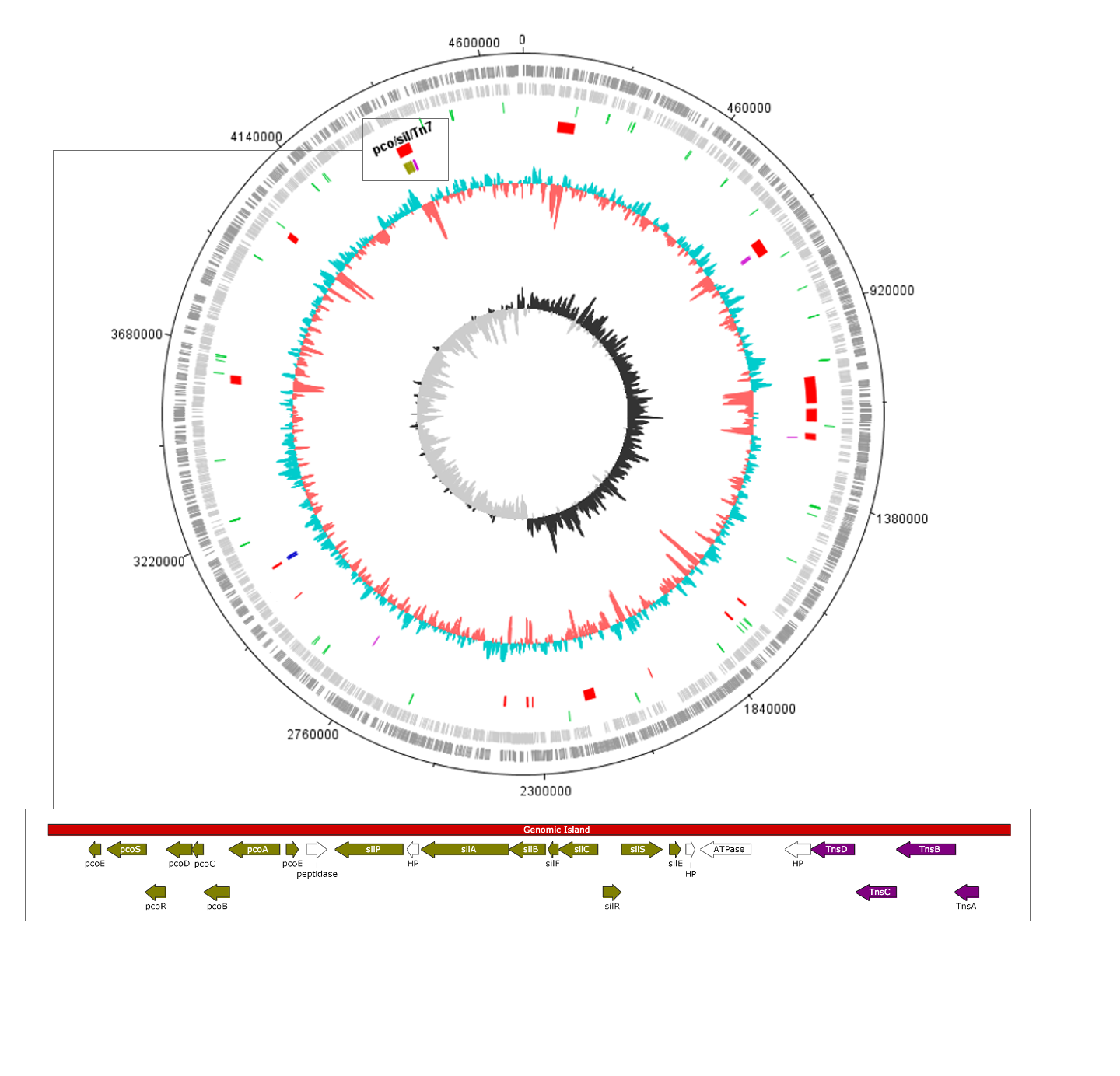

Supplement: Supplementary file 2 — Additional file 2: Figure S1. Chromosomal map of the E. hormaechei isolate CM18-216. From outer to inner circles: 1, nucleotide positions; 2 and 3, CDSs (grey); 4, tRNA (green); 5, predicted genomic islands and prophages (red); 6, pco/sil copper/silver resistance(brown-green), transposases (purple), salmochelin synthesis and uptake (blue); 7, GC% plot; 8, GC skew plot. Inset: detailed map of the pco/sil resistance locus. [file 13756_2020_828_MOESM2_ESM.tiff]

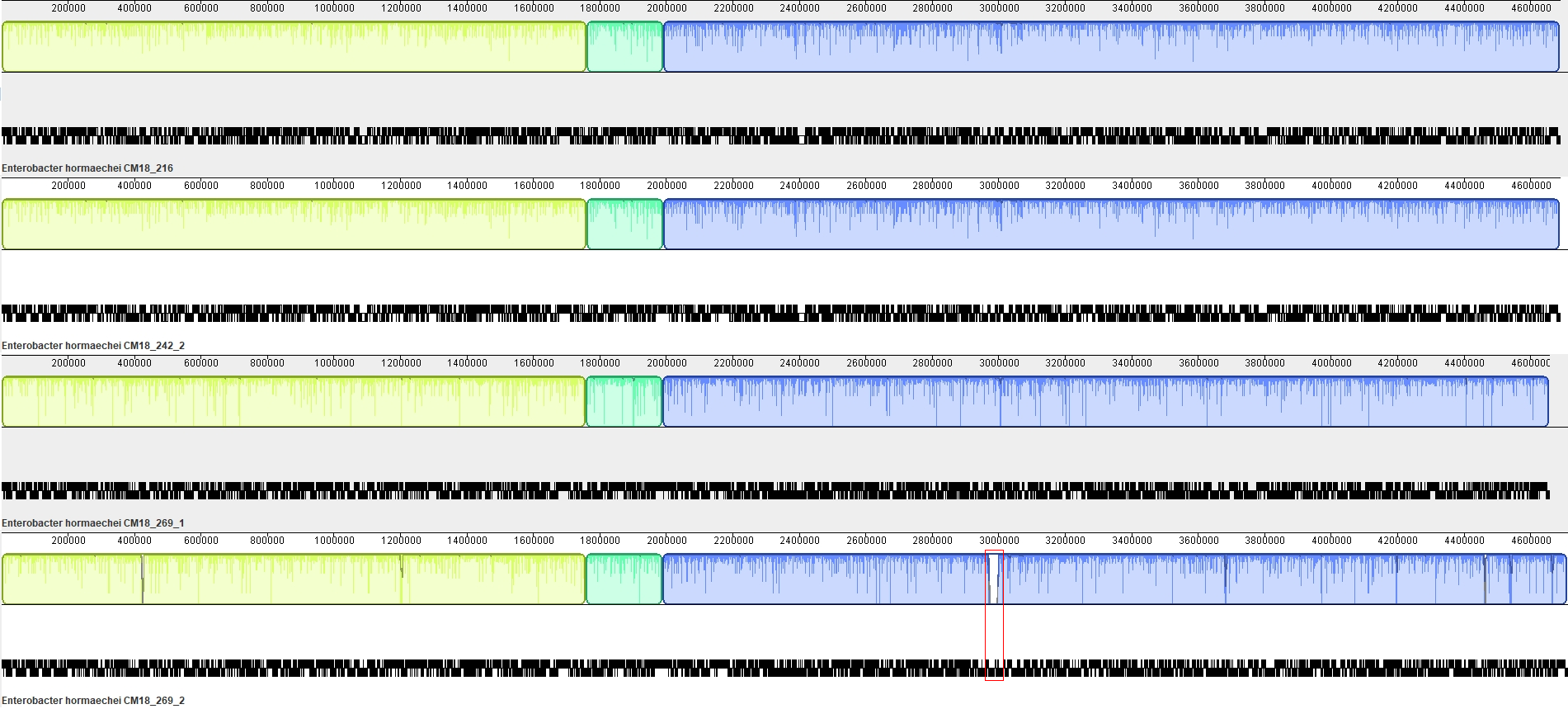

Supplement: Supplementary file 3 — Additional file 3: Figure S2. Mauve alignment of the chromosome sequences of the four Enterobacter strains isolated from the ICU sink over approximately one month. Local blocks of colinearity are labelled with different colors. Predicted coding sequences are indicated underneath each genome. CM18-216 and CM18-242-2 (two top rows) were obtained from hybrid assemblies of Illumina and Nanopore reads; CM18-269-1 and CM18-269-2 (two bottom rows) were assembled from nanopore reads only. Position of a putative phage in CM18-269-2 is indicated by a red box. [file 13756_2020_828_MOESM3_ESM.tiff]

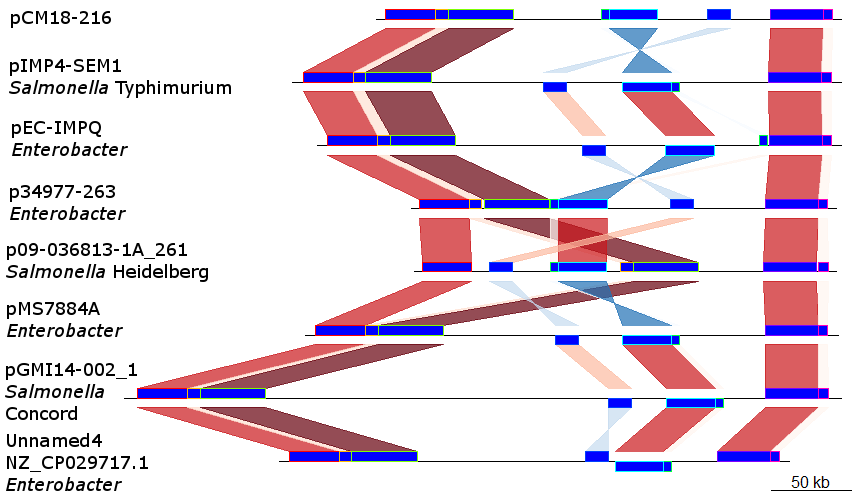

Supplement: Supplementary file 4 — Additional file 4: Figure S3. Alignment of pCM18-216 with IncH12 plasmids carrying qnrB2. Horizontal black lines indicate the lengths of the plasmid sequences. Dark blue horizontal bars on the top (forward strand) and the bottom (reverse strand) of the black lines indicate areas of sequence homology. Vertical bars connecting the horizontal lines show areas of sequence homology. [file 13756_2020_828_MOESM4_ESM.tiff]
